# Health and lifestyle parameters in peripheral artery disease at two periods of the COVID-19 pandemic: comparison between men and women

**DOI:** 10.31744/einstein_journal/2024AO0345

**Published:** 2024-02-19

**Authors:** Hélcio Kanegusuku, Gustavo Oliveira da Silva, Heloisa Amaral Braghieri, Juliana Ferreira de Carvalho, Renan Massena Costa, Gabriel Grizzo Cucato, Nelson Wolosker, Raphael Mendes Ritti-Dias, Marilia Almeida Correia

**Affiliations:** 1 Hospital Israelita Albert Einstein São Paulo SP Brazil Hospital Israelita Albert Einstein, São Paulo, SP, Brazil.; 2 Universidade Nove de Julho São Paulo SP Brazil Universidade Nove de Julho, São Paulo, SP, Brazil.; 3 Northumbria University Newcastle upon Tyne England Northumbria University, Newcastle upon Tyne, England.; 4 Hospital Israelita Albert Einstein Faculdade Israelita de Ciências da Saúde Albert Einstein São Paulo SP Brazil Faculdade Israelita de Ciências da Saúde Albert Einstein, Hospital Israelita Albert Einstein, São Paulo, SP, Brazil.

**Keywords:** COVID-19, Coronavirus infections, Pandemics, Peripheral arterial disease, Social isolation, Intermittent claudication, Sex characteristics, Life style, Exercise, Mental health

## Abstract

**Objective:**

This study analyzed the impact of sex on self-reported health and lifestyle parameters in peripheral artery disease patients at two periods of the COVID-19 pandemic.

**Methods:**

In this longitudinal study, 99 patients with peripheral artery disease (53 men and 46 women) were evaluated during two periods of the COVID-19 pandemic ( *i.e* ., at onset: May to August 2020, and on follow-up: May to August 2021). Patients were interviewed via telephone, and information regarding lifestyle and health parameters was obtained.

**Results:**

At the onset of the COVID-19 pandemic, health and habit parameters were similar between women and men, with 63.0% and 45.3% indicating frequent fatigue, 73.9% and 84.9% reporting increased sitting time, and 23.9% and 39.6% practicing physical activity, respectively. At follow-up, difficulties in physical mobility (women: from 26.1% to 73.9%, p<0.001; men: from 39.6% to 71.7%, p=0.001) and the frequency of hospitalization for reasons other than COVID-19 increased similarly in women and men (women: from 4.3% to 21.7%, p=0.013; men: from 9.4% to 24.5%, p=0.038). The other parameters were similar between the periods.

**Conclusion:**

Self-reported physical mobility difficulties and hospitalization frequency increased in women and men with peripheral artery disease.

## In Brief

While women experience more consequences related to peripheral artery disease than men, such as worse functional capacity and higher morbidity, there was a similar increase in physical mobility difficulty and frequency of hospitalization for reasons other than COVID-19 one year after the onset of the pandemic.

## Highlights

▪Sitting time increased in 73.9% of women and 84.9% of men at the onset of the pandemic.

▪Physical activity was practiced by 23.9% of women and 39.6% of men at the onset of the pandemic.

▪The prevalence of both women and men reporting physical mobility difficulties increased at follow-up.

▪Hospitalization rates for reasons unrelated to COVID-19 have increased in both women and men.

## Graphical abstract

**Figure:**
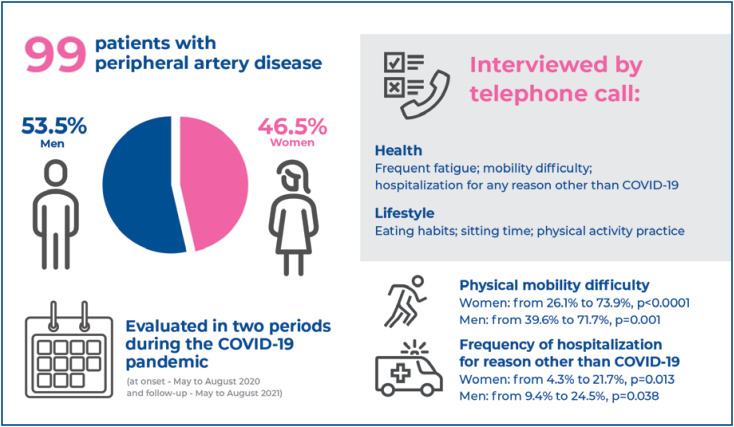

## INTRODUCTION

Social isolation has been one of the main measures adopted to mitigate the spread of coronavirus disease 2019 (COVID-19). The COVID-19 pandemic has negatively impacted lifestyles and health, especially in individuals with long-term conditions, such as those with peripheral artery disease (PAD).^( 1 – 4 )^ Previous studies have reported reduced physical activity levels, increased sedentary behavior, impaired mental health, and decreased walking capacity in PAD patients during the COVID-19 pandemic.^( 1 , 5 – 7 )^

Studies have shown that women suffer more consequences related to PAD than men, presenting with worse functional capacity, impaired vascular function, and higher levels of inflammatory markers and morbidity.^( 8 – 11 )^ Consequently, the prolongation of the COVID-19 pandemic could potentially affect women more than men; however, this remains unclear.

In this study, we investigated lifestyle and health parameters ( *i.e* ., global and physical) during two periods of the COVID-19 pandemic ( *i.e* ., at onset, from May to August 2020; and on follow-up, from May to August 2021) in men and women with PAD. We hypothesized that women with PAD would be more negatively affected by these parameters than men during the COVID-19 pandemic.

## OBJECTIVE

This study analyzed the impact of sex on self-reported health and lifestyle parameters in patients with peripheral artery disease during two periods of the COVID-19 pandemic.

## METHODS

### Study design and participants

This prospective study included patients with PAD recruited from a research database of patients who participated in previous studies by our research group.^( 12 – 14 )^

Patients were included if they met the following criteria: agreed to participate and responded to all survey questions, had a previous diagnosis of PAD; age >45 years. Patients were excluded if they presented with some disability that compromised their answers to the questionnaire during a phone call ( *i.e* ., cognitive, hearing, or speech).

This study was approved by the *Universidade Nove de Julho* Ethics Committee before data collection (CAAE: 31529220.8.0000.5511; # 4.647.088). All patients informed their consent prior to participation.

### Data collection

Data were collected through phone interviews over two time periods: between May and August 2020 (onset) and between May and August 2021 (follow-up). Personal information was retrieved from our database, including demographic data such as sex, date of birth, time from PAD diagnosis (in years), risk factors, and medications.

The impact of sex on longitudinal changes in the self-reported health and habits of patients were assessed using a questionnaire, as previously described.^( 1 , 5 )^ The questionnaire consisted of questions divided into the following domains: overall health, physical health, and habits. In the subsequent section, we present the questions used in our analysis.

Overall health: the participants were required to report the presence of all diagnosed diseases from a prepared list, such as hypertension, diabetes, dyslipidemia, and cardiopathy. In addition, participants were asked if they were hospitalized for any reason other than COVID-19.

Physical health: this domain consists of three self-reported items. Patients were asked the following "yes" or "no" questions: 1) Have you been feeling fatigued in the last few weeks? 2) Have you lost weight in the last few weeks? 3) Have you experienced physical mobility difficulties in the last few weeks?

Habits: to explore the possible impact of COVID-19 on eating habits, the following "yes" or "no" questions were asked: 1) Due to the COVID-19 pandemic, have your eating habits worsened? 2) Due to the COVID-19 pandemic, have you spent more time sitting? 3) Have you practiced physical activity regularly, at least once a week, during the COVID-19 pandemic?

### Statistical analysis

All statistical analyses were performed using SPSS version 20. The Mann–Whitney U test and χ^2^ test were used to compare groups (women and men) for continuous and categorical variables at baseline, respectively. The χ^2^ test was used to compare the differences within each of the groups between the two periods (2020 and 2021) during the COVID-19 pandemic. Data are presented as median and interquartile range for continuous variables and absolute and relative frequencies for categorical variables. Statistical significance was defined as p<0.05.

## RESULTS

One hundred and forty-nine PAD patients participated in this study at the onset of the COVID-19 pandemic. At follow-up, 33 patients could not be contacted, five dropped out for personal reasons, six had a health problem that prevented them from participating in the study, and six died, of which three deaths were due to acute myocardial infarction and one each were due to stroke, kidney disease, and COVID-19. A total of 99 patients (46 women) completed the study. There were no differences between the sexes in terms of age, disease duration, cardiovascular risk factors, or medication use ( Table 1 ).

**Table 1:** Characteristics of peripheral artery disease patients included in the study

Variables		Women (n=46)	Men (n=53)	p value
Age, years		68 (11)	70 (14)	0.300
Disease duration, years		8.5 (7.0)	7.0 (6.0)	0.116
Risk factors, n (%)				
	Diabetes	26 (56.5)	25 (47.2)	0.353
	Dyslipidemia	40 (87.0)	44 (83.0)	0.586
	Hypertension	39 (84.8)	43 (81.1)	0.631
Medications, n (%)				
	ACEi	9 (19.6)	17 (32.1)	0.158
	ARA	15 (32.6)	12 (22.6)	0.267
	Antiplatelet	35 (76.1)	46 (86.8)	0.168
	Beta-blockers	24 (52.2)	20 (37.7)	0.149
	Diuretics	20 (43.5)	21 (39.6)	0.698
	Hypoglycemics	23 (50.0)	22 (41.5)	0.397
	Statins	40 (87.0)	41 (77.4)	0.217
	Vasodilators	17 (37.0)	22 (41.5)	0.644

Data are presented as median (interquartile range) or absolute and relative frequency.

ACEi: angiotensin-converting enzyme inhibitor; ARA: angiotensin receptor antagonist.

The self-reported health and habit parameters were also similar between sexes at the onset of the COVID-19 pandemic ( Table 2 ).

**Table 2:** Self-reported health parameters evaluated in women and men with peripheral artery disease at the onset of the COVID-19 pandemic

Variables		Women (n=46)	Men (n=53)	p value
Physical health, n (%)				
	Frequent fatigue	29 (63.0)	24 (45.3)	0.077
	Weight loss	11 (23.9)	12 (22.6)	0.881
	Mobility difficulty	12 (26.1)	21 (39.6)	0.154
Overall health, n (%)				
	Hospitalization for any reason other than COVID-19	2 (4.3)	5 (9.4)	0.325
Habits, n (%)				
	Worsening eating habits	9 (19.6)	5 (9.4)	0.149
	Increased sitting time	34 (73.9)	45 (84.9)	0.174
	Physical activity	11 (23.9)	21 (39.6)	0.096

Data are presented as absolute and relative frequencies.

**Figure 1:**
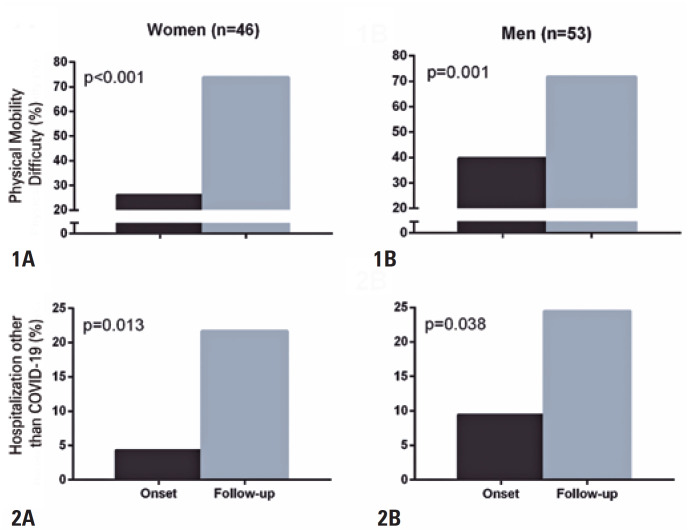
Self-reported physical mobility difficulty and frequency of hospitalization for reasons other than COVID-19 evaluated in women and men with peripheral arterial disease at study onset and follow-up during the COVID-19 pandemic

The self-reported physical mobility difficulty (women: from 26.1% to 73.9%, p<0.001; men: from 39.6% to 71.7%, p=0.001) and frequency of hospitalization for reasons other than COVID-19 (women: from 4.3% to 21.7%, p=0.013; men: from 9.4% to 24.5%, p=0.038) increased in both women and men during the study follow-up compared to the onset ( Figure 1 ).

In both women and men, self-reported physical health parameters, specifically frequent fatigue (women: from 63.0% to 60.9%, p=0.830; men: from 45.3% to 58.5%, p=0.174) and weight loss (women: from 23.9% to 32.6%, p=0.354; men: from 22.6% to 22.6%, p=1.000), and self-reported lifestyle parameters, specifically worsening eating habits (women: from 19.6% to 6.5%, p=0.063; men: from 9.4% to 7.5%, p=0.727), increased sitting time (women: from 73.9% to 60.9%, p=0.182; men: from 84.9% to 77.4%, p=0.321), and physical activity (women: from 23.9% to 19.6%, p=0.613; men: from 39.6% to 30.2%, p=0.308), were similar between study onset and follow-up.

## DISCUSSION

This study demonstrated that self-reported health and lifestyle parameters were similar between women and men with PAD at the onset of the COVID-19 pandemic. After one year of the COVID-19 pandemic, self-reported health and lifestyle parameters remained similar to those at onset, except for self-reported physical mobility difficulty and frequency of hospitalization for reasons other than COVID-19, which increased in women and men with PAD.^( 8 – 11 )^

Studies^( 8 , 9 , 14 )^ performed before the COVID-19 pandemic showed that women with PAD had worse health ( *e.g* ., physical mobility indicators) and lifestyle ( *e.g* ., lower physical activity levels and more sedentary behavior) parameters than men with PAD. However, contrary to this evidence, with the exception of a trend towards increased sitting time in men than women with PAD (84.9% *versus* 73.9%, p=0.174), other self-reported health and lifestyle parameters were similar between men and women in the baseline assessment performed within the first few months of social isolation in Brazil. The findings regarding sitting time may be related to the fact that 30.2% of the men and 23.9% of the women stopped engaging in physical activity during the first few months of the COVID-19 pandemic. In general, studies^( 1 , 5 – 7 )^ performed on patients with PAD, but without analyzing the sexes separately, during the onset of social isolation demonstrated reduced walking capacity, deteriorating mental health, increased sedentary behavior, and lower physical activity levels compared to the period before the COVID-19 pandemic. A possible explanation is that in women with PAD, many of these parameters were already significantly altered before the COVID-19 pandemic and changed little during the onset of the pandemic. In contrast, those in men may have significantly worsened, reaching values similar to those in women.

Regarding the longitudinal impact of the COVID-19 pandemic, most self-reported health ( *i.e* ., frequent fatigue and weight reduction) and lifestyle *(i.e* ., eating habits, physical activity level, and sedentary behavior) parameters did not change after a year in both women and men with PAD. This result contradicts those of other studies^( 15 – 18 )^ performed in adults without PAD that observed changes in eating habits, weight gain, a reduction in physical activity levels, and an increase in sedentary behavior during the COVID-19 pandemic. In patients with PAD, the possible impact of social isolation at the onset of the COVID-19 pandemic on these parameters, which had significantly been altered previously, may have mitigated the changes a year after.

Men and women with PAD reported similar increases in physical mobility difficulties and hospitalizations for reasons other than COVID-19. Some studies before the COVID-19 pandemic observed that PAD severity, comorbid conditions, and social support ( *i.e* ., marital status) were predictors of physical function in PAD patients^( 19 – 21 )^ and that non-cardiovascular ( *e.g* ., respiratory problems, cellulitis, sepsis and other infections, and *diabetes mellitus* ) and cardiovascular causes ( *e.g* ., heart failure and chronic ischemic heart disease) were responsible for 68% and 32% of hospitalizations in patients with PAD, respectively.^( 22 )^ These factors and conditions may have worsened in men and women with PAD during their longitudinal social isolation in Brazil. It is important to highlight that the causes of hospitalization one year after the onset of the COVID-19 pandemic are unclear in subjects with or without PAD. Studies^( 23 – 26 )^ performed on adult subjects without PAD and COVID-19 from different countries during the COVID-19 pandemic ( *i.e* ., until August 2020), but without comparing the sex, observed that falls, respiratory disease, stroke, cardiac diseases ( *i.e* ., acute myocardial infarction and heart failure), kidney disease, oncological diseases, and metabolic/endocrine diseases have been the causes of hospitalization. This suggests that the same phenomenon may have also occurred longitudinally ( *i.e* ., between May and August 2021) in patients with PAD.

This situation is worrying because in addition to the associated comorbidities, specifically peripheral atherosclerosis in patients with PAD, limb ischemia has been significantly more serious during hospitalization, leading to an increased rate of perioperative complications compared to the period before the COVID-19 pandemic.^( 27 )^ In this context, the results of the present study demonstrate the importance of multidisciplinary care for women and men with PAD during the COVID-19 pandemic. Telemedicine consultations, telerehabilitation programs, and unsupervised home-based exercise programs, among other strategies, are potential options for improving the overall health (physical and mental health, healthy eating habits, and physical activity) of these patients, regardless of sex.

This study has some limitations. First, the use of self-reported assessments is susceptible to information bias. Additionally, subjective assessments ( *e.g* ., 6-minute walk test) were not performed due to the social isolation adopted to contain the spread of COVID-19. Lastly, these results cannot be extrapolated to other populations with other characteristics.

## CONCLUSION

The self-reported health and habit parameters were similar between women and men at the onset of the COVID-19 pandemic. After one year, women and men with peripheral arterial disease showed similar increases in self-reported physical mobility difficulty and frequency of hospitalization for reasons other than COVID-19.

## References

[1] Ritti-Dias RM, Correia MA, Carvalho JF, Braghieri HA, Wolosker N, Cucato GG (2022). Impact of the COVID-19 pandemic on health lifestyle in patients with peripheral artery disease: a cross-sectional study. J Vasc Nurs.

[2] Guarinello GG, D’Amico RC, Miranda AN, Novack J, Coral FE (2022). Impact of COVID-19 on the surgical profile of vascular surgery patients at a tertiary hospital in Curitiba, Brazil. J Vasc Bras.

[3] Donato G, Pasqui E, Alba G, Abu Leil M, Palasciano G (2021). The limitations of social behaviour imposed by covid-19 impacted the perception and the evolution of peripheral arterial disease negatively. Ann Vasc Surg.

[4] Sena G, Gallelli G (2020). An increased severity of peripheral arterial disease in the COVID-19 era. J Vasc Surg.

[5] Ritti-Dias RM, Cucato GG, Oliveira MD, Braghieri HA, Carvalho JF, Wolosker N (2021). Physical activity practice during COVID-19 pandemic in patients with intermittent claudication. Rev Assoc Med Bras (1992).

[6] Lanzi S, Pousaz A, Buso G, Mazzolai L, Calanca L (2021). Partial home confinement during the COVID-19 pandemic, physical function, and physical activity in patients with symptomatic lower extremity peripheral artery disease. Vasc Med.

[7] Braghieri HA, Correia MA, Carvalho JF, Longano P, Wolosker N, Cucato GG (2021). Impact of the COVID-19 pandemic on drug treatment of patients with peripheral arterial disease: an observational cross-sectional study. J Vasc Bras.

[8] Gardner AW, Parker DE, Montgomery PS, Sosnowska D, Casanegra AI, Ungvari Z (2015). Gender and racial differences in endothelial oxidative stress and inflammation in patients with symptomatic peripheral artery disease. J Vasc Surg.

[9] Gardner AW, Parker DE, Montgomery PS, Khurana A, Ritti-Dias RM, Blevins SM (2010). Gender differences in daily ambulatory activity patterns in patients with intermittent claudication. J Vasc Surg.

[10] Correia MA, Sousa AS, Andrade-Lima A, Germano-Soares AH, Zerati AE, Puech-Leao P (2020). functional and cardiovascular measurements in patients with peripheral artery disease: comparison between men and women. J Cardiopulm Rehabil Prev.

[11] Hiramoto JS, Katz R, Ix JH, Wassel C, Rodondi N, Windham BG, Harris T, Koster A, Satterfield S, Newman A, Shlipak MG, Health ABC study (2014). Sex differences in the prevalence and clinical outcomes of subclinical peripheral artery disease in the Health, Aging, and Body Composition (Health ABC) study. Vascular.

[12] Braghieri HA, Kanegusuku H, Corso SD, Cucato GG, Monteiro F, Wolosker N (2021). Validity and reliability of 2-min step test in patients with symptomatic peripheral artery disease. J Vasc Nurs.

[13] Kanegusuku H, Cucato GG, Domiciano RM, Longano P, Puech-Leao P, Wolosker N (2020). Impact of obesity on walking capacity and cardiovascular parameters in patients with peripheral artery disease: a cross-sectional study. J Vasc Nurs.

[14] Sousa AS, Correia MA, Farah BQ, Saes G, Zerati AE, Puech-Leao P (2019). Barriers and levels of physical activity in patients with symptomatic peripheral artery disease: comparison between women and men. J Aging Phys Act.

[15] Fernández-Abascal EG, Martín-Díaz MD (2022). One year of COVID-19 in Spain, longitudinal study on mental and physical health. Behav Med.

[16] Goncalves A, Le Vigouroux S, Charbonnier E (2021). University Students’ lifestyle behaviors during the COVID-19 pandemic: a four-wave longitudinal survey. Int J Environ Res Public Health.

[17] Bhutani S, vanDellen MR, Cooper JA (2021). Longitudinal Weight Gain and Related Risk Behaviors during the COVID-19 Pandemic in Adults in the US. Nutrients.

[18] Lueger-Schuster B, Zrnić Novaković I, Lotzin A (2022). Two Years of COVID-19 in Austria-Exploratory Longitudinal Study of Mental Health Outcomes and Coping Behaviors in the General Population. Int J Environ Res Public Health.

[19] Oka RK, Szuba A, Giacomini JC, Cooke JP (2004). Predictors of physical function in patients with peripherial arterial disease and claudication. Prog Cardiovasc Nurs.

[20] Farah BQ, Souza Barbosa JP, Cucato GG, Chehuen MR, Gobbo LA, Wolosker N (2013). Predictors of walking capacity in peripheral arterial disease patients. Clinics (Sao Paulo).

[21] Chen X, Stoner JA, Montgomery PS, Casanegra AI, Silva-Palacios F, Chen S (2017). Prediction of 6-minute walk performance in patients with peripheral artery disease. J Vasc Surg.

[22] Arruda-Olson AM, Moussa Pacha H, Afzal N, Abram S, Lewis BR, Isseh I (2018). Burden of hospitalization in clinically diagnosed peripheral artery disease: a community-based study. Vasc Med.

[23] Sultanoğlu H, Demir MC, Boğan M (2022). Trends in Geriatric Trauma Emergency Department Admissions During COVID-19. J Trauma Nurs.

[24] Yang Y, Le KJ, Liang C, Zheng T, Gu ZC, Lin HW (2022). Changes in inpatient admissions before and during COVID-19 outbreak in a large tertiary hospital in Shanghai. Ann Transl Med.

[25] Morello F, Bima P, Ferreri E, Chiarlo M, Balzaretti P, Tirabassi G (2021). After the first wave and beyond lockdown: long-lasting changes in emergency department visit number, characteristics, diagnoses, and hospital admissions. Intern Emerg Med.

[26] Kuitunen I, Ponkilainen VT, Launonen AP, Reito A, Hevonkorpi TP, Paloneva J (2020). The effect of national lockdown due to COVID-19 on emergency department visits. Scand J Trauma Resusc Emerg Med.

[27] Trunfio R, Deslarzes-Dubuis C, Buso G, Fresa M, Brusa J, Stefanescu A (2021). The effects of COVID-19 pandemic on patients with lower extremity peripheral arterial disease: a near miss disaster. Ann Vasc Surg.

